# The role of active soil carbon in influencing the profitability of fertilizer use: Empirical evidence from smallholder maize plots in Tanzania

**DOI:** 10.1002/ldr.3940

**Published:** 2021-04-05

**Authors:** Jordan Chamberlin, T. S. Jayne, Sieglinde Snapp

**Affiliations:** ^1^ Socioeconomics Program CIMMYT‐Kenya Nairobi Kenya; ^2^ Department of Agricultural, Food, and Resource Economics Michigan State University East Lansing Michigan USA; ^3^ Department of Plant, Soil, and Microbial Sciences Michigan State University East Lansing Michigan USA

**Keywords:** active carbon, fertilizer response, maize, smallholder agriculture, soil fertility

## Abstract

We use recent plot‐level panel data from Tanzanian smallholder farmers to investigate maize yield responses to inorganic fertilizer under variable soil carbon conditions. Unlike many prior studies which consider total carbon measurements, we focus on active soil carbon, which is a component strongly related to key soil functions, such as nutrient cycling and availability. Active soil carbon is found to be a strong predictor of maize yield response to nitrogen fertilizer. These results highlight important sources of variation in the returns to fertilizer investments across plots and smallholder farmers in the region. When farmgate prices for maize and fertilizer are incorporated into calculations of economic returns, we find that the profitability of fertilizer use is strongly dependent upon farmgate price ratio assumptions: under our most optimistic agronomic response estimates, 39% of farmer plots have an average value‐cost ratio greater than 1.5 at a maize‐nitrogen price ratio of 0.15. That share drops to 4% at a price ratio of 0.12 and 0% at a price ratio of 0.09. Our findings provide insights into the intertwined biophysical and economic underpinnings of low levels of fertilizer use in Tanzania and elsewhere in the region. Raising active carbon stocks in smallholder systems may be a strategic priority in many areas for incentivizing greater use of inorganic fertilizer, reversing land degradation, and achieving sustainable agricultural intensification.

## 1 MOTIVATION

Staple crop yields in subSaharan Africa (SSA) remain very low by international standards, with yield gaps on the order of 80% (van Ittersum et al., 2016). Inorganic fertilizer is widely agreed to be the technology with the greatest potential to raise yields in SSA's smallholder systems (Holden, 2018; Vanlauwe et al., 2014). Inorganic fertilizer also greatly promotes crop biomass and is therefore an important component of an integrated and sustainable soil fertility management strategy.

Nitrogen is the main constraining nutrient for cereal crop performance across most environments, both in terms of yield level and yield stability (Vanlauwe et al., 2011). Indeed, nitrogen has been identified as one of the grand challenges of the 21st century given its pivotal role in food production, and nowhere is this more important than in subSaharan Africa where a strong negative relationship has been observed between soil nitrogen balances and population density (Drechsel, Gyiele, Kunze, & Cofie, 2001). Yet the relatively low uptake of nitrogen fertilizers by SSA smallholders indicates important constraints, which are not yet fully understood.

The large spatial heterogeneity in fertilizer usage in SSA (Sheahan & Barrett, 2017) suggests that both market factors (e.g., farmgate crop/fertilizer price ratios) as well as environmental factors (such as soil and rainfall) may play an important role. Yield response—that is, the marginal or average physical product of fertilizer—is often low and highly variable for smallholder staples producers, resulting in low levels of profitability of fertilizer use when farmgate crop and fertilizer prices are applied (Burke, Jayne, & Black, 2017; Jayne & Rashid, 2013; Koussoubé & Nauges, 2017; Liverpool‐Tasie, Omonona, Sanou, & Ogunleye, 2017; Marenya & Barrett, 2009; Sheahan, Black, & Jayne, 2013; Theriault, Smale & Haider, 2018; Xu, Guan, Jayne, & Black, 2009).^1^ Relatedly, fertilizer is not profitable for many farmers even where the *average* benefits are positive and relatively large, given differences in management ability and other factors that vary across plots and households (Suri, 2011). Fertilizer responses in many areas may be limited by depleted soil organic matter (Drechsel et al., 2001; Marenya & Barrett, 2009), soil acidity (Burke et al., 2017), and other factors. Risk‐averse farmers are especially likely to forgo expected gains in the face of uncertainty around the performance or profitability of a given technology (Emerick, de Janvry, Sadoulet, & Dar, 2016; Magruder, 2018). Disentangling the patterns of fertilizer responses may help us to better understand how to design area‐, household‐, and plot‐specific interventions to overcome constraints to the profitable use of fertilizer in African smallholder production systems.

This study identifies key soil‐related drivers of maize yield and maize yield response to nitrogen fertilizers for Tanzanian smallholders. Our particular emphasis is on organic carbon, a particularly important component of soil fertility (Lal, 2006; Nord & Snapp, 2020). We use two‐wave panel data on farmer‐managed plots in 25 maize‐producing districts in Tanzania. In addition to the standard farmer‐, farm‐ and community‐level characteristics typically included in such analyses, our dataset features well‐measured yields (through yield subplot crop cuts at harvest time), plot‐level soil chemical analysis, and detailed plot‐level agronomic management information. We find that estimated maize yield response to N is similar to other empirical studies from the region based on farmer‐managed fields and that they are strongly conditioned by both rainfall and active soil carbon. Our production function estimates indicate that the marginal product (i.e., agronomic efficiency^2^) of nitrogen increases by 16–21% when moving from the 25th to the 75th percentile of active carbon in our sample. Furthermore, the variability around these expected returns are high. After factoring in local input and output prices, profitability assessments indicate relatively low returns to fertilizer investments: less than half of the sample have an average value cost ratio (AVCR) >1.5 under our most favorable estimation results and very conservative estimates of farmgate price ratios. Our results also highlight differences in conclusions about the profitability of fertilizer use on farmers' own fields and management conditions versus studies relying on farm trials and demonstration plots (e.g., Jama, Kimani, Harawa, Mavuthu, & Sileshi, 2017), which tend to benefit from researcher management protocols that many smallholder farmers may not be able to replicate (Snapp, Jayne, Mhango, Benson, & Ricker‐Gilbert, 2014). Our results highlight the importance of considering the factors that condition fertilizer response (and profitability) from the farmer's standpoint when designing agricultural intensification programs and investment strategies. Our analysis concludes that agricultural intensification strategies based on raising the intensity of fertilizer use are unlikely to lead to widespread adoption if the variation in agronomic and economic returns is not accounted for and if the sources of low active soil carbon are not also addressed.

The rest of this article is organized as follows. After describing our setting, data and empirical estimation strategy, we present estimation results for agronomic and economic returns to fertilizer investments, in turn. We discuss these results and their implications for sustainable intensification strategies, concluding with key messages for policymakers and recommendations for further research.

## 2 EMPIRICAL FRAMEWORK

### 2.1 Context

Tanzania is one of the largest countries in Eastern and Central Africa, and an important source of the region's maize production. However, most of this production comes from smallholders who have relatively low levels of productivity, and few of which use modern inputs such as fertilizer. As such, raising maize yields has been an important investment and policy target for the country and its partners in recent years. Tanzania is representative in many ways of the maize‐based farming systems found elsewhere in the region, in terms of its agroecologies and range of biophysical endowments, the predominant production characteristics of its smallholder farmers, and the relatively low levels of market infrastructure development. At the same time, the heterogeneity of production characteristics found within Tanzania's maize growing areas bodes well for its value as a test case for evaluating variability of agronomic responses across key geographical characteristics (Nord & Snapp, 2020).

### 2.2 Data

**FIGURE 1:**
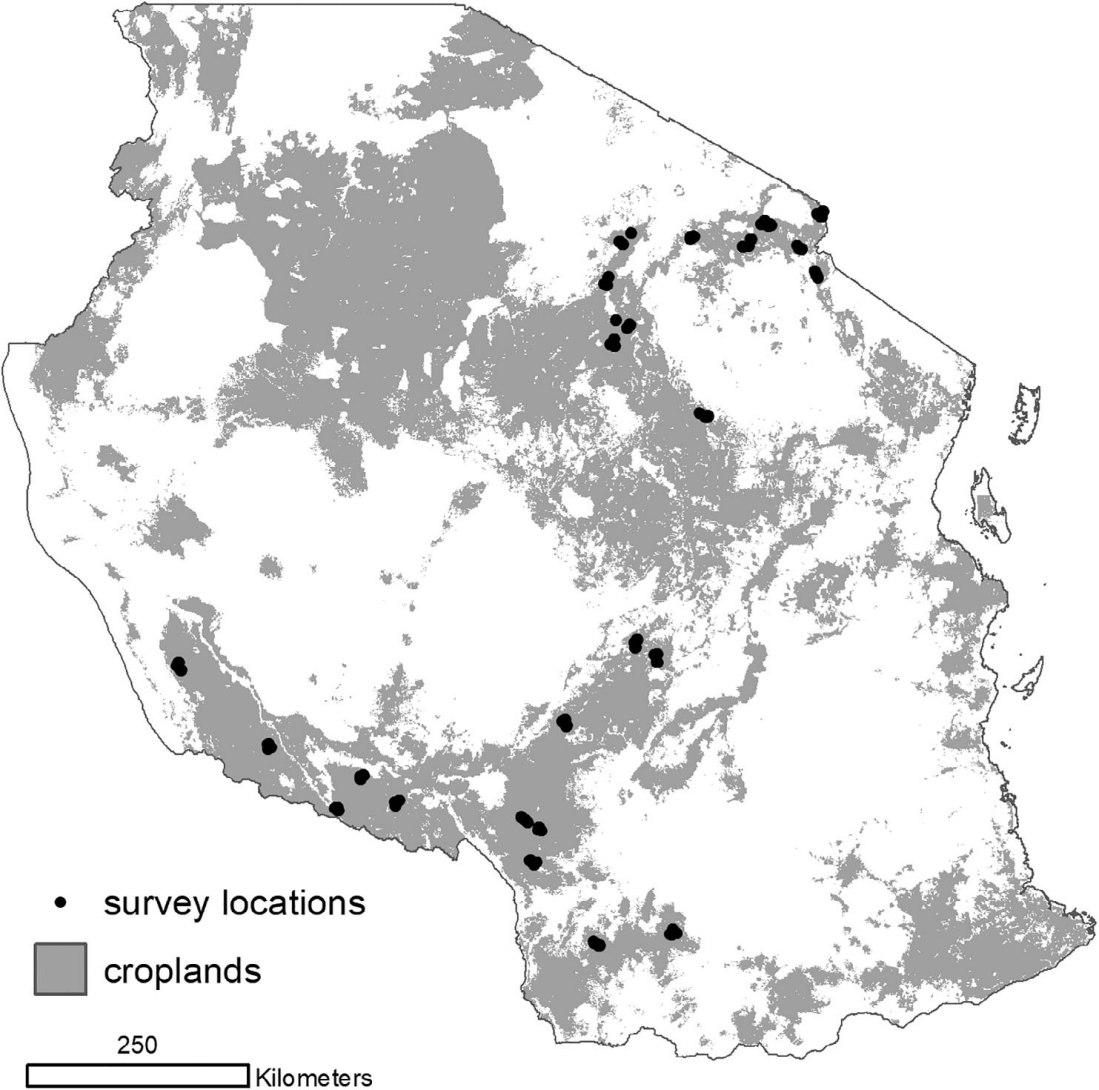
Survey locations

Farm household survey data were collected in Tanzania in 2016 and 2017 on 624 households, located in 25 districts (Figure 1). These districts are located in both the Southern Highlands and Northern Zone, representing the most important maize growing areas in the Country. Within each district, a stratified sampling frame was used that maximized soil type variability so as be able to make broad inferences about crop response, and to identify survey localities (Shepherd, Shepherd, & Walsh, 2015; Walsh & Vågen, 2006). Within each locality, a listing of all maize producing households was generated with the assistance of the local headman. From this listing, 24 households in each locality were randomly selected. Data were collected on household demographics, farm and nonfarm economic portfolios, land holdings and productive assets, and other characteristics. Within each farm household, basic information was collected for each plot managed by the household (e.g., land use status, production decisions). In addition, very detailed agronomic management information was collected for household's most important maize plot (henceforth the farm's 'focal plot'). This plot was identified by the farmer as the plot which generated the most maize production, and which received the most managerial effort.

Nitrogen and other macronutrient supplies were calculated from the various fertilizer blends farmers reported using. We drop observations with fertilizer application rates exceeding 700 kg ha^−1^, as implausible and likely to derive from data entry error.^3^

Maize yields on focal plots were measured using crop cuts from three 5 × 5 meter quadrants, calculated at 12.5% grain moisture content. Soil characteristics from these plots were measured from samples taken at quadrant locations at 0–10 and 10–20 cm depths.

Total organic carbon, despite its well‐recognized importance as an indicator of overall soil quality, is not an ideal indicator of nutrient availability because much of the bulk soil organic matter is relatively inert (Drinkwater, Wagoner, & Sarrantonio, 1998). Soil organic carbon is largely conditioned by topography and soil parent material; however, once a field is converted to agriculture, active soil organic matter fractions largely determine soil productivity, and this is markedly influenced by farmer practices (Zingore, Delve, Nyamangara, & Giller, 2008). Thus, rather than testing for total carbon, as is often the case in standardized soil testing, testing the active organic matter pool provides better insight into how changes in management affect nutrient cycling and potential soil carbon accumulation or loss (Haynes, 2005; Wander, 2004). The active carbon pool, while constituting a small fraction (5–20%) of the soil's total organic matter, is the component that greatly influences key soil functions, such as nutrient cycling and availability, soil aggregation, and soil carbon accumulation (Grandy & Robertson, 2007; Schmidt et al., 2011; Six, Elliott, Paustian, & Doran, 1998; Wander, 2004). Hence, in this analysis, we focus on the factors influencing active carbon.

Developments in laboratory assays to monitor 'active' soil organic matter fractions have highlighted the value of permanganate oxidizable carbon (POXC) as an early indicator of management influence on soil organic carbon (Culman et al., 2012). We acknowledge that there are other laboratory analyses that track this fraction. At the same time, POXC is a widely used indicator and we standardize for the purposes of this study on the term 'active carbon' to refer to POXC results. Total soil organic carbon also provides insights regarding sustainable soil management, although at a slow timestep (5–10 years). For this work, POXC was determined on a ground (1 mm sieve) subsample, oxidized with 0.02 M KMnO_4_, and subsequently absorbance was read of the liquid phase at a wavelength of 550 nm (Culman et al., 2012). POXC (mg kg^−1^) = [0.02 M − (*a* + *b* × Abs)] × (9,000 mg C mol^−1^) × (0.02 L solution/Wt) where 0.02 M is KMnO_4_ solution initial concentration, *a* is the intercept, *b* is the standard curve slope, Abs is the absorbance of the unknown soil sample, 9,000 mg is the amount of C oxidized by 1 mol of MnO_4_ changing from Mn^7+^ to Mn^2+^, 0.02 L is the volume of KMnO_4_ solution reacted, and Wt is the mass of soil (kg) (Weil, Islam, Stine, Gruver, & Samson‐Liebig, 2003). To address potential measurement error in our analysis, and under the assumption that the soil properties of interest here are comparatively stable in the very short term, we use the average measure across the two consecutive years for each plot in our regression work.

Rainfall was measured as the sum of dekadal values recorded for the main growing season, using the CHIRPS dataset (Funk et al., 2017). Rainfall variability was measured as the coefficient of variation on the dekadal observations within a season.

### 2.3 Estimation strategy

The intent of this article is to understand the agronomic and economic returns to nitrogen fertilizer applications in smallholder maize production. In keeping with agronomic and agricultural economic literature, we frame maize yield (y) as a function of fertilizer application rates (F), other agronomic management decisions (M), and other exogenous conditioners (G):

(1) $$y=f\left(F,M,G\right).$$

Because farmers in Tanzania use a variety of fertilizer blends, we integrate these decisions be decomposing each blend into its macronutrient content, that is, nitrogen (N), phosphorus (P) and potassium (K). Other management factors include improved maize seed, maize–legume intercropping (common in the southern highlands), organic matter integration via compost, manure and crop residue retention, plant spacing, weeding, fallowing, terracing and erosion control structures, and herbicide and pesticide applications. Other exogenous conditioners include slope, rainfall, rainfall variability and the presence of disease or striga (*striga asiatica*, a.k.a. witchweed).

We adopt a flexible polynomial functional form, allowing for quadratic terms and interactions between variables. In this approach, we follow similar empirical studies (e.g., Burke et al., 2017; Sheahan et al., 2013; Xu et al., 2009). This flexibility is important in enabling us to investigate how yield response to nitrogen is conditioned by other factors. We may generalize this function as:

(2) $$y_{\mathrm{it}}=\alpha +\beta_{1}N_{\mathrm{it}}+\beta_{2}N_{it}^{2}+\beta_{10}X_{\mathrm{it}}++\beta_{11}N_{\mathrm{it}}\times X_{\mathrm{it}}+u_{\mathrm{it}},$$

*Where:* N is nitrogen, our primary input of interest, *i* indexes plots, *t* indexes observations over time, and where, for convenience, we have subsumed **M** and **G** in the vector **X**. As indicated earlier, a priori hypotheses include the possibility of positive interactions between nitrogen, soil organic carbon and rainfall, after controlling for other factors.^4^

A key consideration is the possibility that unobserved factors may possibly bias our estimation results. Concretely, we may decompose the residual in Equation (2) as:

(3) $$u_{\mathrm{it}}=o_{\mathrm{it}}+c_{i}+\epsilon_{\mathrm{it}},$$

*Where:* *o* represents unobserved time‐varying factors, *c* represents unobserved time‐constant factors, and *ϵ* is a randomly distributed error term. Time‐varying unobservables may include soil moisture, nutrient status or other factors which are often missing from empirical studies (or poorly measured). Time‐constant unobservables may include farmer ability or plot biophysical characteristics which change little from year to year, but which may affect both fertilizer usage and yield outcomes. Finally, correlation between model covariates and the stochastic error term may be an additional source of bias.^5^

In the present study, we argue that our dataset does a better job at controlling for time‐varying plot and plot management factors than is typically the case in empirical studies, and therefore unobserved *o*_*it*_is unlikely to be a major issue. Our larger concern is with time‐invariant unobserved farmer and plot‐level heterogeneity which are likely to upwardly bias our results if not addressed (e.g., under the assumption that more able farmers are more likely to use fertilizer than less able farmers). To address this, we estimate models with the Mundlak–Chamberlain device (i.e., the Correlated Random Effects model (Wooldridge, 2010), as well as a fixed effects estimator.

## 3 RESULTS

### 3.1 Descriptive statistics

Summary statistics on our dataset are reported in Table 1. The average farm size is 3.3 ha, and is comprised of four plots. Most of our sample consists of farms in the 1–4 ha range, which is typical for smallholder systems in the region. Only 7% had a single plot, and 14% had more than five plots. The mean and median focal plot sizes are 0.85 and 0.51 ha, respectively. A total of 13% of our sample farms are managed by female household heads.

**TABLE 1:** Summary statistics of sample

Variable	Units	25th	50th	75th	Mean
Farm/farmer characteristics					
Farm size	ha	1.11	2.02	3.64	3.30
# of plots	count	2	3	4	4
Focal plot size	ha	0.26	0.51	1.03	0.85
Household size	Members	4	5	7	6
Farmer age	Years	38	48	59	49
Farmer education	Years	8	8	8	7
Female	Binary	—	—	—	0.13
Focal plot characteristics					
Yield	kg ha^−1^	1,112	2,748	4,464	2,996
Used fertilizer	Binary	—	—	—	0.32
N (for fertilizer users)	kg ha^−1^	38	60	101	84
P (for fertilizer users)	kg ha^−1^	0	10	55	38
K (for fertilizer users)	kg ha^−1^	0	0	0	3
Active carbon	mg kg^−1^	337	481	696	534
Total carbon	mg kg^−1^	4,717	6,869	10,604	8,818
Topsoil pH	pH	5.6	6.1	6.5	6.1
Intercropped	Binary	—	—	—	0.54
Legume rotation	Binary	—	—	—	0.09
Compost	Binary	—	—	—	0.01
Manure	Binary	—	—	—	0.19
Crop residues retained	Binary	—	—	—	0.06
Herbicide	Binary	—	—	—	0.01
Pesticide	Binary	—	—	—	0.01
Improved seed	Binary	—	—	—	0.33
N. weedings	Count	1	2	2	1.57
Disease	Binary	—	—	—	0.12
Striga	Binary	—	—	—	0.03
Fallowed w/in 3 years	Binary	—	—	—	0.05
Erosion control structures	Binary	—	—	—	0.14
Terraced	Binary	—	—	—	0.04
Sloped	Binary	—	—	—	0.77
Rainfall	mm	322	383	759	528
Rainfall CV	CV	0.49	0.65	0.85	0.66

*Note:* Summary statistics on farmer characteristics and management decisions calculated for the 553 observations recorded in year 2017. Statistics on carbon measures calculated on the pooled sample

Yields in our sample are somewhat higher than the national averages reported elsewhere for Tanzania, with a median value of 2.7 tons ha^−1^. This reflects the fact that the focal plot is not a random maize plot, but the most important and generally most productive plot available to the farmer. Furthermore, because our sample districts were selected on the basis of being important maize producing districts, maize yields in our sample likely reflect more favorable production conditions than a nationally representative sample. This sample orientation notwithstanding, only about a third of sample uses fertilizer on these plots.^6^ Of these fertilizer users, there is considerable variability in fertilizer application rates, with a median rate of 60 kg ha^−1^ of nitrogen (somewhat below regional recommendations).^7^

### 3.2 Agronomic returns to nitrogen

Production function coefficient estimates are shown in Table 2 (we show estimation results for the variables of primary interest in this discussion; full results are reported in Table S1). We show six alternative specifications. In each of these, the dependent variable is maize yield, measured in kg ha^−1^ during the maize production season. Nitrogen, as expected, shows a strong positive and nonlinear influence on yield outcomes. Specifications (1) and (2) use pooled OLS (POLS), and only differ in the interaction term: the first specification interacts N with active carbon alone, while the second specification interacts N with active carbon and log rainfall for that growing season. Specifications (3) and (4) incorporate the Mundlak–Chamberlain device—that is, the correlated random effects (CRE) model—to address unobserved heterogeneity, but are otherwise similar to the first two specifications. Specifications (5) and (6) use Fixed Effects estimation to address unobserved heterogeneity, but are otherwise similar to the other specification pairs. All models are cluster robust at the household level and include controls for plot and household characteristics, detailed plot management controls, rainfall and rainfall variability, a year indicator, and, in the POLS and CRE models, time‐invariant controls for the 75 districts in the sample.

**TABLE 2:** Production function estimates

Variables	(1)	(2)	(3)	(4)	(5)	(6)
	POLS	POLS	CRE	CRE	FE	FE
N	9.329***	9.069***	8.482***	8.214**	10.31**	9.797**
	(2.874)	(2.884)	(3.195)	(3.207)	(4.781)	(4.812)
N^2^	−0.0141**	−0.0142**	−0.0122**	−0.0123**	−0.0125	−0.0134
	(0.00605)	(0.00604)	(0.00613)	(0.00612)	(0.0114)	(0.0110)
POXC	0.484	0.474	0.491	0.480		
	(0.342)	(0.342)	(0.343)	(0.343)		
N × POXC	0.00440**		0.00432**		0.00545*	
	(0.00172)		(0.00189)		(0.00298)	
N × POXC × log(rain)		0.000775**		0.000765**		0.00109**
		(0.000325)		(0.000356)		(0.000525)
Log(rain)	2,990***	2,998***	3,782***	3,796***	3,853***	3,879***
	(894.1)	(894.1)	(1,060)	(1,060)	(1,139)	(1,137)
CV(rain)	−1,357*	−1,356*	−1,207	−1,204	−1,890**	−1,867*
	(804.5)	(804.3)	(835.9)	(836.1)	(959.3)	(957.8)
Estimator	POLS	POLS	CRE	CRE	FE	FE
HH, farm, and plot controls	Yes	Yes	Yes	Yes	Yes	Yes
Plot management controls	Yes	Yes	Yes	Yes	Yes	Yes
Mundlak–Chamberlain controls	No	No	Yes	Yes	No	No
District dummies	Yes	Yes	Yes	Yes	No	No
Year dummies	Yes	Yes	Yes	Yes	Yes	Yes
Observations	599	599	599	599	498	498
*R*‐squared	0.332	0.332	0.344	0.344	0.238	0.239

*Note:* The dependent variable in all models is maize yield measured in kg ha^−1^. Rainfall measured in 10‐day periods during the growing season for the survey year. *SEs* are cluster robust at the household level. Full results shown in Table S1

Abbreviations: CV, coefficient of variation; N, nitrogen; POXC, active carbon

* *p* < .1

** *p* < .05

*** *p* < .01

Coefficient estimates (Table 2) are fairly consistent across all specifications, although they differ somewhat in magnitude. Results correspond with the expected positive returns to N applications, but at diminishing rates. Interaction terms—N × POXC and N × POXC × log(rainfall)—are significant under all three estimators, indicating that the agronomic efficiency of N is conditioned by active carbon and rainfall, as hypothesized. The coefficients on active carbon and its interaction term is highly significant in all models, even where the individual coefficient for active carbon is not significant at conventional levels.^8^ The estimated impacts of rainfall and rainfall variability are positive and negative, respectively, as we would expect.

Average marginal effects are shown for N and POXC in Table 3. The marginal effects for N are our estimates of marginal product (MP).^9^ These estimates differ somewhat across specifications, being somewhat higher under FE compared with POLS and CRE models. The range in MP estimates of 10–13 (additional kgs of maize yield per additional kg of N) are similar to those found elsewhere in the region: 8 kg in Nigeria (Liverpool‐Tasie et al., 2017), 16 kg for Zambia (Xu et al., 2009), 17 kg for Kenya (Marenya & Barrett, 2009), 23–25 kg for Uganda (Matsumoto & Yamano, 2013), 21–25 kg for Malawi (Harou, Liu, Barrett, & You, 2017), 19 kg for Burkina Faso (Koussoubé & Nauges, 2017). Our results are somewhat higher than Mather, Minde, Waized, Ndyetabula, and Temu (2016) found for Tanzania using LSMS‐ISA data (7–8 kg). However, their data included all plots and production in marginal areas, and was based on farmer estimates, rather than crop‐cut measures. Because our sample focuses on the most productive maize plots of farmers in Tanzania's maize producing belt, we would expect somewhat higher levels of productivity than for the entire population of smallholders in the Nation.

**TABLE 3:** Partial effects of nitrogen and active carbon (estimates from fixed effects models)

	(1)	(2)	(3)	(4)	(5)	(6)
	POLS	POLS	CRE	CRE	FE	FE
N	10.87***	10.82***	10.08***	10.04***	12.49***	12.60***
	(2.741)	(2.734)	(3.065)	(3.059)	(4.458)	(4.404)
POXC	0.607*	0.614*	0.611*	0.618*	0.150*	0.193**
	(0.329)	(0.328)	(0.330)	(0.330)	(0.0818)	(0.0932)
Interaction	N × POXC	N × POXC × log(rain)	N × POXC	N × POXC × log(rain)	N × POXC	N × POXC × log(rain)

*Note:* Table shows partial effects of nitrogen (N) and active carbon (POXC) from model results in Table 2. Units are kg maize per kg N and kg maize per mg kg^−1^ POXC, respectively. *SEs* are cluster robust at the household level. *SEs* are cluster robust at the household level.

* *p* < .1

** *p* < .05

*** *p* < .01

In the analysis that follows, we focus on the results of the fixed effects regression, as the model which has the most plausible controls for unobserved time‐invariant heterogeneity which may otherwise bias our results. However, we may note that all our results (i.e., limited agronomic and economic returns to fertilizer) are similar across all model specifications.

Table 4 illustrates the diminishing expected MP of nitrogen at different concentrations of active carbon (10th, 25th, 50th, 75th, and 90th percentile, respectively), holding other factors constant. Focusing on the Fixed Effects model results, which are arguably most conservative, the direct impact of moving from 337 mg kg^−1^ (the 25th percentile of our sample) to 696 mg kg^−1^ (75th percentile) implies an increase in MP by 17–21 percentage points, depending upon the specification (i.e., whether or not log rainfall enters via an interaction). Moving from the 10th to the 90th percentile of the active carbon distribution is associated with even larger changes in MP: 37–47 percentage points. POLS and CRE results are very similar.

**TABLE 4:** Partial effects of nitrogen at different levels of active carbon in sample (estimates from fixed effects models)

	(1)	(2)	(3)	(4)	(5)	(6)
	POLS	POLS	CRE	CRE	FE	FE
MP at POXC = 220 (10th pctile)	9.517***	9.344***	8.756***	8.579***	10.83**	10.55**
	(2.611)	(2.606)	(2.950)	(2.944)	(4.296)	(4.286)
MP at POXC = 337 (25th pctile)	10.03***	9.904***	9.257***	9.131***	11.46***	11.34***
	(2.648)	(2.638)	(2.981)	(2.970)	(4.336)	(4.305)
MP at POXC = 481 (50th pctile)	10.66***	10.60***	9.880***	9.819***	12.24***	12.31***
	(2.715)	(2.707)	(3.041)	(3.033)	(4.423)	(4.374)
MP at POXC = 696 (75th pctile)	11.61***	11.64***	10.81***	10.84***	13.42***	13.76***
	(2.852)	(2.861)	(3.172)	(3.185)	(4.624)	(4.566)
MP at POXC = 954 (90th pctile)	12.75***	12.88***	11.92***	12.07***	14.82***	15.51***
	(3.068)	(3.117)	(3.387)	(3.447)	(4.965)	(4.921)
ΔMP: 25th–75th POXC pctile	16%	18%	17%	19%	17%	21%
ΔMP: 10th–90th POXC pctile	34%	38%	36%	41%	37%	47%
Interaction	N × POXC	N × POXC × log(rain)	N × POXC	N × POXC × log(rain)	N × POXC	N × POXC × log(rain)

*Note:* Table shows partial effects of nitrogen at 10th, 25th, 50th, 75th, and 90th percentile of active carbon in the sample, from the estimation results reported in Table 2. Units are kg maize per kg N at different concentrations of POXC. *SEs* are cluster robust at the household level

* *p* < .1;

** *p* < .05

*** *p* < .01

Given the uncertainty that farmer face in production environments, these expected changes in MP are not at all trivial. Recall that rainfall variability also affects response. Because rainfall is a stochastic variable, the large impact it has on yields, even after controlling for other factors, indicates the magnitude of uncertainty in yield outcomes for farmers operating in these areas.

As a complement to our MP estimate, we computed the average physical product (AP) of N, calculated as the difference between the estimated difference in yields resulting from zero fertilizer and yields resulting from 250 kg ha^−1^ of nitrogen (the level at which MVCR = 1, on average, when using a farmgate maize‐nitrogen price ratio of 0.15), with other sample values as observed. The distribution of MP and AP estimates across the sample is shown in Table 5. These results indicate substantial variability in agronomic response across the sample. As an illustration, a farmer at the 75th percentile of the MP distribution has an expected MP that 28% larger than that of a farmer at the 25th percentile.

**TABLE 5:** Distribution of MP and AP estimates

Physical product	10th	25th	50th	75th	90th	Mean	*SD*
MP	8.69	10.20	11.62	13.03	14.42	11.37	2.82
AP	8.11	8.78	9.80	11.18	12.27	9.98	1.76

*Note:* MP and AP estimates come from the fixed effects model corresponding to column 6 in Table 2. MP is calculated for each household using sample values. MP is measured as kg maize per kg N. AP is calculated as the estimated difference in grain yield (kg ha^−1^) resulting from zero fertilizer and the yield resulting from 250 kg ha^−1^ of nitrogen (the level at which MVCR = 1, on average, when using a farmgate maize‐nitrogen price ratio of 0.15), with other sample values as observed

Abbreviations: AP, average physical product; MP, marginal product

### 3.3 Economic returns to nitrogen

To translate these agronomic responses into profitability terms, we calculate and summarize a number of relative measures of economic returns on the basis of alternative maize‐nitrogen price ratios. In our farm survey data, the farmer‐reported input and output prices were exceedingly noisy and it was not possible to coherently interpret the variability of responses within a given area. Data entry problems cannot be ruled out, but we may also note the wide variety of fertilizer acquisition and maize sales channels: farmers buy and sell at very different quantities, in different types of markets, at different distances from their homestead. For this reason, we base the profitability analysis in this article on a set of representative wholesale prices based on different sources of local market price information for Tanzania: data on the average maize wholesale prices in regional markets was taken from FEWSNet for the 2014–2018 period. Data on the average unsubsidized commercial price of urea (generally the cheapest source of N) for all local Tanzanian markets reporting prices for 50 kg bags during the 2014–2018 period was obtained AfricaFertilizer.org. The price for nitrogen was inferred from the urea price, based on the 46% N content of urea, as is standard practice in this type of analysis. Based on these data, we define a representative market price ratio, as well several indicative farmgate price ratios (Table 6). The representative maize/nitrogen market price ratio of 0.22, based on 0.27 and 1.22 USD kg^−1^ for maize and nitrogen, respectively. These values are very similar to those used in other studies of fertilizer profitability for Tanzanian maize farmers (e.g., Kihara et al., 2016). However, such a market price ratio fails to account for last mile transfer costs incurred by farmers, in which effective prices of inputs increase (as the farmer needs to add transport costs to the market price paid) and the effective prices of marketed output decline (as the farmer must discount transfer costs between the farm and the market from the market price received).^10^ Thus, we further define farmgate price ratios from the baseline market price ratio, based on transfer costs of 0.006 $US/kg km^−1^ at 5, 10, 15, and 20 km distance, respectively, between the wholesale market and the farmgate, resulting in decreasing price ratios of 0.18, 0.15, 0.12, and 0.09. The transfer cost assumption here is based on the empirical finding of Benson, Kirama, and Onesmo (2012). Our resulting farmgate price ratios are in the range of those calculated by Mather et al. (2016) from LSMS‐ISA data for Tanzania (which range from 0.19 to 0.14).

**TABLE 6:** Maize and N price assumptions used in profitability calculations

Price ratios used for profitability analysis	Maize ($US/kg)	Nitrogen ($US/kg)	Maize/N price ratio
Wholesale price ratio: 0.22	0.27	1.22	0.22
Farmgate price ratio: 0.18	0.24	1.33	0.18
Farmgate price ratio: 0.15	0.21	1.44	0.15
Farmgate price ratio: 0.12	0.18	1.55	0.12
Farmgate price ratio: 0.09	0.15	1.66	0.09

The marginal value‐cost ratio (MVCR) is computed as the MP multiplied by the input–output price ratio, while the average value‐cost ratio (AVCR) is the AP multiplied by the input–output price ratio. An AVCR value exceeding 1 indicates profitability, strictly speaking, although an AVCR value of 2 is often used as a shorthand criterion for gauging the economic attractiveness of an investment from the perspective of a risk‐averse farmer. Similarly, while an MVCR value of 0 indicates the optimal input level for a risk‐neutral farmer (because marginal returns are zero), MVCR values of 1 or greater are often used as more reasonable indicators of acceptable minimum marginal returns, under assumptions of risk‐aversion and imperfectly observed production or transactions costs.

Table 7 summarizes these measures for the different price ratio assumptions, using the estimation results from the FE model with N × POXC × log(rainfall) interactions (column 6 in Table 2). This specification produces the highest estimated agronomic response of maize to N. As such, these results may be taken as an upper bound to the actual profitability of fertilizer in our survey area.

**TABLE 7:** Profitability distributions under alternative maize‐N price ratios

MVCR	10th	25th	50th	75th	90th	Mean	*SD*	MVCR>0 (%)	MVCR>1 (%)	MVCR>2 (%)
Wholesale price ratio: 0.22	1.92	2.26	2.57	2.88	3.19	2.52	0.62	99	97	87
Farmgate price ratio: 0.18	1.57	1.84	2.10	2.35	2.60	2.05	0.51	99	96	60
Farmgate price ratio: 0.15	1.27	1.49	1.69	1.90	2.10	1.66	0.41	99	96	18
Farmgate price ratio: 0.12	1.01	1.18	1.35	1.51	1.67	1.32	0.33	99	90	1
Farmgate price ratio: 0.09	0.79	0.92	1.05	1.18	1.30	1.03	0.25	99	60	0

AVCR	10th	25th	50th	75th	90th	Mean	*SD*	AVCR>1 (%)	AVCR>1.5 (%)	AVCR>2 (%)
Wholesale price ratio: 0.22	1.79	1.94	2.17	2.47	2.71	2.21	0.39	100	98	69
Farmgate price ratio: 0.18	1.46	1.58	1.77	2.02	2.21	1.80	0.32	99	87	26
Farmgate price ratio: 0.15	1.18	1.28	1.43	1.63	1.79	1.46	0.26	98	39	2
Farmgate price ratio: 0.12	0.94	1.02	1.14	1.30	1.42	1.16	0.20	80	4	0
Farmgate price ratio: 0.09	0.73	0.79	0.89	1.01	1.11	0.90	0.16	26	0	0

*Note:* Calculations based on the MP and AP estimates shown in Table 5, against each of the price ratio assumptions in Table 6

Abbreviations: AVCR, average value‐cost ratio; MVCR, marginal value‐cost ratio

Results indicate relatively low rates of profitability, regardless of the assumption: the average MVCR ranges from 2.52 (at the market price ratio of 0.22) to 1.03 (when the farmgate price ratio is 0.09). While most farmers apply at rates below the economically efficient rate for a risk‐neutral farmer (i.e., where MVCR = 0), the share of farmers with MVCR > 1 drops notably with price ratio reductions, and the share of farmers with MVCR > 2 drops even faster. As discussed elsewhere (e.g., Morris, Kelly, Kopicki, & Byerlee, 2007; Sheahan et al., 2013; Xu et al., 2009), an MVCR of two or more has conventionally been used by agricultural economists as the level of returns required to induce fertilizer adoption by smallholders, based on empirical observations of fertilizer use patterns in the region. However, as Morris et al. (2007) also note, in especially risky production contexts, value‐cost ratios of 3 or 4 may be required to incentive adoption by smallholders.

AVCR estimates show similar cross‐sectional variability, with mean values ranging between 2.21 (at price ratio = 0.22) to 0.90 (at price ratio = 0.09). It is common to use an AVCR of 1.5 or 2 as a minimal threshold of profitability sufficient to incentivize risk‐averse smallholder farmers to use fertilizer, to account for risk aversity and unobserved transactions costs in production and marketing (e.g., Sheahan et al., 2013; Xu et al., 2009). The share of farmers in the sample with AVCR estimates exceeding 1.5 or 2 is very sensitive to price ratio assumptions: at a price ratio of 0.15 only 39 and 2% of our sample has an AVCR exceeding 1.5 and 2, respectively. Our results suggest that under even moderate uncertainty about farm gate prices, the magnitude of the MVCR and AVCR estimates may be insufficient to motivate farmers to make risky fertilizer investments. There is a growing body of evidence from the region that modest MVCR and AVCR returns to fertilizer use on maize is the norm for smallholder agriculture (Burke et al., 2017; Sheahan et al., 2013). This could in large part be due to agronomic practices that are ineffective at maintaining soil productivity or adequate weed control (Burke, Snapp, & Jayne, 2020).

**FIGURE 2:**
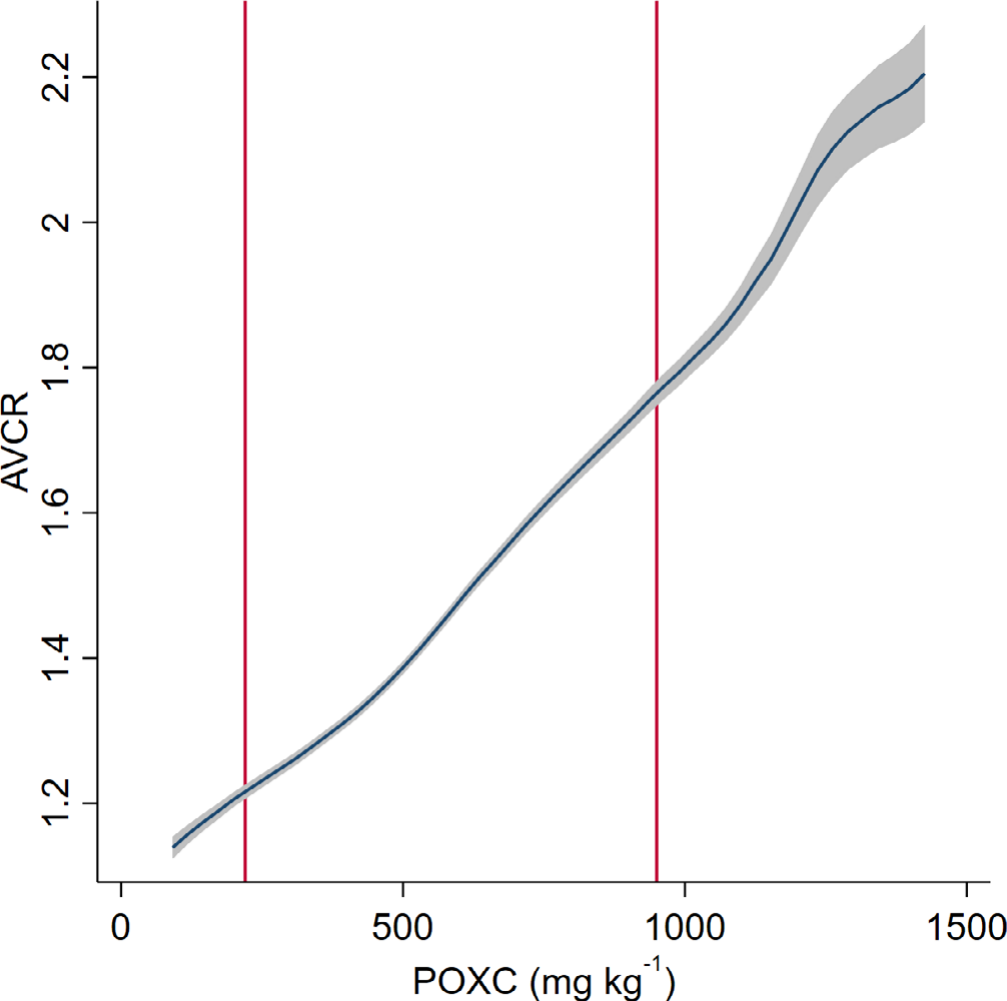
Estimated average value‐cost ratio (AVCR) over distribution of active carbon in sample. Vertical red lines indicate 10% and 90th percentiles of the distribution of POXC measures in the sample. AVCR estimates use AP estimates from the fixed effects model with N × POXC × log(rainfall) interaction (column 6 in Table 2), and a farmgate maize‐nitrogen price ratio of 0.15. POXC is measured as mg kg^−1^ [Colour figure can be viewed at wileyonlinelibrary.com]

**FIGURE 3:**
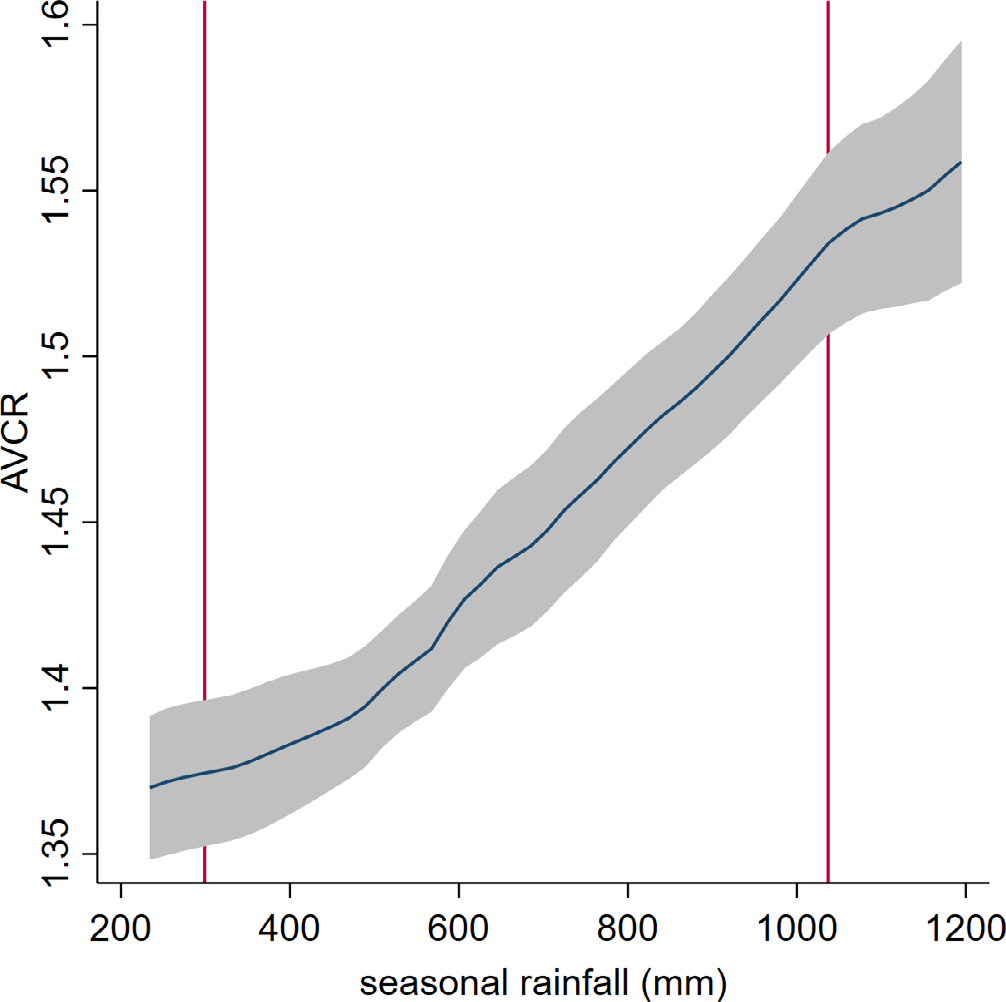
Estimated average value‐cost ratio (AVCR) over distribution of seasonal rainfall in sample. Vertical red lines indicate 10% and 90th percentiles of the distribution of seasonal rainfall totals in the sample. AVCR estimates use AP estimates from the fixed effects model with N × POXC × log(rainfall) interaction (column 6 in Table 2), and a farmgate maize‐nitrogen price ratio of 0.15 [Colour figure can be viewed at wileyonlinelibrary.com]

Further, these findings suggest that even where agronomic returns are positive and of magnitudes generally considered conducive to investment, the incorporation of 'last mile' transportation costs may quickly attenuate the economic attractiveness of these investments (e.g., Minten, Stifel, & Koro, 2013). The implications of economic remoteness have been well described (e.g., Chamberlin & Jayne, 2013; Stifel & Minten, 2008). Adding uncertainty around the actual costs of last mile transportation (which is the reality for many farmers in rural Tanzania) will only magnify the disincentivizing effects of these transfer costs on fertilizer investments. The fact that active soil carbon is an empirically important driver of agronomic responses may help to target attention to where these market remoteness effects may be especially magnified. Figure 2 shows the AVCR calculated at a price ratio of 0.15 as a nonparametric function of active carbon. This graph illustrates that at lower concentrations of active carbon the agronomic use efficiency of nitrogen is likely to be insufficient to be an attractive investment for risk‐averse farmers, even in average rainfall years. When we additionally consider the estimated impacts on profitability of seasonal rainfall (Figure 3), we can clearly see the sensitivity of expected profitability calculations to stochastic factors.

## 4 DISCUSSION

Our results indicate that while the marginal and average agronomic returns to inorganic fertilizer use are generally positive, there are strong variations in these returns over our sample. We have shown that both early season rainfall and active soil carbon are important conditioners of yield responses to nitrogen. The active soil carbon as indicated by POXC has been shown to be correlated with soil nitrogen availability, and vigorous maize growth (Culman, Snapp, Gentry, & Green, 2013). Building on these agronomic response estimates, our economic analysis has stark implications. Even under relatively modest assumptions of last mile transportation costs, the inclusion of estimated farmgate prices in relative profitability calculations reduces the attractiveness of fertilizer investments for a large share of our sample.

One caveat to our analysis is the fact that we do not observe long‐term historical patterns of management. A plot which has received relatively high levels of organic and inorganic fertilizers in the past may benefit from the residual effects of those soil amendments, as well as have higher levels of organic carbon (Njoroge, Schut, Giller, & Zingore, 2019). While we cannot entirely rule out this possibility, our panel estimation framework does address time‐invariant unobserved heterogeneity as well as effects that change slowly over time, which may include cumulative nutrient buildup.^11^

On balance, the findings we present in this article are likely to overestimate Tanzanian smallholders' agronomic responses to fertilizer use and hence their economic incentives to use fertilizer, for several reasons. First of all, our sample consists of farmers in Tanzania's maize belt, where agroecological conditions are generally more favorable than in most other parts of the country. Secondly, the fact that this analysis is based on focal plots, rather than on all plots, means that our analysis cannot be taken as representative of all maize production conditions, but rather of preferential conditions within the smallholder maize system. Farmer preferential allocation of maize crops to higher fertility, adequate soil organic carbon status field has been well documented (Mhango, Snapp, & Kanyama‐Phiri, 2013; Tittonell, Vanlauwe, Corbeels, & Giller, 2008). As such, our estimates of agronomic and economic returns to fertilizer use are likely an upward bound on the true values for the system. Problems with acute soil organic carbon depletion and other soil fertility issues are likely to be much worse on average over the farming system as a whole.

Thirdly, our analysis uses the most favorable production function estimates, that is, those resulting from the Fixed Effects estimation. Profitability analysis using the POLS and CRE estimators is even less profitable on average (although in most other respects estimation results are remarkably consistent). When we rerun the same economic analysis using the estimates of agronomic returns generated from the POLS and CRE estimation results, the share of farmers for with MVCR and AVCR estimates exceeding 2 is even lower.

Fourth, our estimates of farmgate maize/nitrogen price ratios, upon which profitability estimates critically depend, are conservative and likely to overestimate the ratio for many farmers. Our price ratio assumptions are somewhat higher than those used in other empirical analysis in the region. Sheahan et al. (2013) find an average maize/nitrogen price ratio of 0.083 for Kenya. Matsumoto and Yamano (2013) find ratios of 0.063–0.075 in Kenya and 0.044–0.027 in Uganda. The price ratio assumptions we employ in our analysis are probably optimistic; many farmers in Tanzania are likely to regularly face less favorable farmgate price ratios. As such, our profitability analysis is most likely biased upwards.

Finally, our results highlight important sources of uncertainty in both agronomic and economic returns to fertilizer investments. We see this particularly in the role of seasonal rainfall and rainfall distribution parameters in the production functions, but may also note that the large uncertainty around input, output and transportation prices faced by farmers means that calculating expected returns on fertilizer investments is highly uncertain even under optimal biophysical production contexts. The fact that active soil carbon has such a strong effect on yield responses in our sample is all the more striking given these considerations. What this means is that even for the most productive smallholders, the agronomic and economic returns to fertilizer use are quite variable, which would further impede the incentives of risk‐averse farmers to incur high capital outlays on fertilizers.

Taken together, these results indicate that efforts to spur fertilizer usage by smallholder farmers in Tanzania should not focus exclusively on blanket agronomic targets, which are based on average responses over large areas, but rather should carefully consider localized response rates. This is in alignment with conclusions from other studies, for example, Nord and Snapp (2020), who studied soil fertility variability in the same geography. Investments in extension and promotion of integrated use of organic management practices in combination with fertilizer are urgently required if maize based systems are to be productive, which has clear implications for reform of input‐based agricultural subsidies (Adolwa, Schwarze, & Buerkert, 2019; Lal, 2006). New technologies that can measure active carbon using handheld spectral devices on farmer's fields will move this project forward, as information will be provided in real time using nonlaboratory, inexpensive methods (TerAvest, Austic, Tu, & Snapp, 2019).

Current agricultural policies often miss the mark, as there is growing evidence—including this study—regarding the accumulation of active soil carbon as being necessary to raise yield responses sufficiently for nitrogen fertilizer to become economically attractive. This may be particularly valid for risk adverse farmers in areas facing high transport costs to regional input and output markets. Failure to address these issues may continue to stall the process of sustainably raising fertilizer use on the majority of Africa's smallholder farms. There is rising urgency in this challenge, as the closure of the land frontier in many African farming areas has led to more frequent continuous cropping of plots, which, without greater usage of fertilizers, will certainly contribute to land degradation and rural poverty (Barbier & Hochard, 2012). Our analysis aligns with other assessments of soil carbon depletion as a major soil degradation concern within the region's smallholder farming systems (Lal, 2006). There is need for more evidence regarding smallholder farm practices that are feasible to adopt, and that increase active soil carbon. Long‐term field experimentation has shown that promising options include crop residues and mixed cropping systems that provide enhanced vegetative cover, although economic assessments have been limited to date (Beedy, Snapp, Akinnifesi, & Sileshi, 2010; Cong et al., 2015).

Success in investing in soil organic carbon can also help mitigate the variability of yields in the face of highly variable weather and a changing climate (Williams et al., 2016). Our analysis suggests that agronomic returns to nitrogen will have to increase substantially in order to offset the low and variable price margins that smallholder farmers typically face in countries like Tanzania.

## 5 CONCLUSIONS

Many areas of smallholder cultivation in subSaharan Africa have been systematically experiencing reductions in soil organic carbon levels, particularly where continuous maize cultivation without legume rotations and/or application of inorganic or organic fertilizer is common (Drechsel et al., 2001; Lal, 2006; Marenya & Barrett, 2009; Shepherd & Soule, 1998; Tittonell, Vanlauwe, Leffelaar, Shepherd, & Giller, 2005). Low soil organic carbon stocks are known to affect plant uptake of nutrient supplies and figure into low responsiveness of yields to nitrogen supply in some areas. Farmers in such areas are much less likely to find fertilizer investments to be economically attractive, regardless of how well fertilizer markets are performing. Because of its role in promoting greater crop biomass (including roots), inorganic fertilizer is usually a crucial component of a sustainable agricultural intensification strategy, and its use will need to rise rapidly to arrest the land degradation processes already evident in areas where the land frontier has been reached and where continuous cultivation has become the norm.

This article has assembled evidence on the ways in which maize yield responses to inorganic fertilizer are affected by soil organic carbon and other factors. We have shown that the agronomic efficiency of nitrogen is positively associated with both soil organic carbon stocks (measured as active carbon), as well as by seasonal rainfall. When farmgate prices for maize and fertilizer are incorporated into calculations of average and marginal cost–benefit ratios, we find that economic returns—as measured by MVCR and AVCR—drop drastically over price ratio ranges that are representative of those likely faced by many smallholders in Tanzania. For example, assuming a price ratio of 0.15, most farmers cannot utilize fertilizer profitably (39% have an estimated AVCR > 1.5, and only 2% have an estimated AVCR > 2). However, even under more favorable assumptions, the high year‐to‐year variability around these expected returns would discourage many farmers from investing in fertilizer even if it were profitable to do so on average across all years. Our results indicate that the sensitivity of fertilizer profitability to such outcome uncertainty will be particularly acute in areas of depleted organic matter.

An important implication of our analysis is the importance of investments and policy interventions which address the systematic depletion of soil organic carbon stocks. This study adds to the growing evidence that cereal crop response to fertilizer requires that attention to be paid to soil health, as indicated by active soil carbon. There have been previous calls by soil scientists and economists for integrated soil fertility management to complement inorganic fertilizer, yet few previous studies have examined this at scale. Our research, conducted across a broad range of soil types and market contexts across Tanzania, reasserts the urgency of this proposition to better inform discussions of how to stimulate fertilizer investments by African smallholders. Farmer incentives to make such investments will be promoted by efforts to raise the agronomic efficiency of fertilizer sufficiently for fertilizer to be profitable.

## CONFLICT OF INTEREST

The authors declare no conflicts of interest.

## Supporting information


**Table S1**. Full regression results for production function estimates (corresponds with Table 2).
**Table S2**. Production function estimates using total carbon (instead of POXC).
**Table S3**. Marginal effects from production function estimates using total carbon (instead of POXC).

## Data Availability

Data used in this study are available from https://data.cimmyt.org/.

## Endnotes

^1^ See Jayne, Mason, Burke, and Ariga (2018) for a recent review of over 20 studies estimating the profitability of applying inorganic fertilizer on maize in various African locations.

^2^ The change in crop output resulting from an additional unit of an input (such as nitrogen fertilizer) is referred to by economist as *marginal product* or *marginal physical product*; in the agronomy literature this is referred to as *agronomic efficiency*.

^3^ However, we may note that this does not affect our results qualitatively. We obtain similar results from models run on the entire sample, models run on the sample with outliers dropped, and models run on the sample with winsorized outliers. In the case of winsorizing, we replaced N application rates exceeding 700 kg ha^−1^ with that value, which is tantamount to winsorizing at the 99th percentile of N application rates for fertilizer users, and which follows the protocols used by Liverpool‐Tasie et al. (2017) and Sheahan and Barrett (2017). However, as noted, these outlier management choices do not materially affect our analytical conclusions.

^4^ Our assumption of positive interactions between nitrogen, soil organic carbon and rainfall is grounded in the context of no excessive rainfall, which was the case in our study. However, it should be acknowledged that such interactions may be negative at excessively high levels of rainfall. Such interactions may also be negative under conditions of very high organic matter content, especially when the latter derives from nitrogen fixing trees or excessive green manuring. The latter conditions are also unlikely to be the case in our study sample. We thank an anonymous reviewer for raising this issue.

^5^ Burke et al. (2017) provide a useful, detailed discussion of these issues and corresponding identification strategies in survey data settings.

^6^ Sheahan and Barrett (2017) estimate that 17% of Tanzanian farm households use fertilizer, drawn from the nationally representative 2011 wave of the LSMS‐ISA data. Mather et al. (2016), using three waves of the LSMS‐ISA data, find similar national‐level estimates, but note higher levels of fertilizer use by maize farmers in the zones covered by our survey: 31–37% of maize plots in the Southern Highlands and 16% of maize plots in the Northern Zone.

^7^ Sheahan and Barrett (2017) found that fertilizer users applied an average of 32 kg ha^−1^ in the nationally representative LSMS‐ISA data for Tanzania in 2011. The higher application rates we find for fertilizer users in our sample reflects our sample design, as noted above, as well as the fact that fertilizer use in our sample is dominated by high analysis urea (46%N) and was often applied to very small maize plots.

^8^ For comparison, we also estimated models which used total carbon instead of POXC. Coefficient estimates and marginal effects of N and carbon from these models are shown in Tables S2 and S3. The results with total carbon show a smaller direct association with carbon on yield outcomes and show less significant interaction effects with yield response to N, although the direct yield responses to N in these models were otherwise very similar. It is possible that in some other empirical settings, well‐measured estimates of total carbon may give useful insights into interaction effects, although in our analysis it proved to be of little analytical value, despite the positive correlation between total carbon and active carbon.

^9^ As noted earlier, MP is equivalent to agronomic efficiency.

^10^ Note that this situation does not change when marketing is done locally via traders. In that case, the trader's margins will include transfer costs between the village and market, plus intermediation fees.

^11^ Furthermore, when we control for year‐specific measures of active carbon in our fixed effects model, results indicate interaction effects which are larger in magnitude although less precise than the interaction effects estimated from specifications using the time‐averaged measures of active carbon. This suggests that that lagged effects are not an issue.
