# Supplementary material for: The role of active soil carbon in influencing the profitability of fertilizer use: Empirical evidence from smallholder maize plots in Tanzania
Source: Land Degrad Dev. 2021 Apr 5;32(9):2681–94. doi: 10.1002/ldr.3940 (PMC8251585; doi:10.1002/ldr.3940)
Supplement: Supplementary file 1 — Table S1. Full regression results for production function estimates (corresponds with Table 2). Table S2. Production function estimates using total carbon (instead of POXC). Table S3. Marginal effects from production function estimates using total carbon (instead of POXC). [file LDR-32-2681-s001.docx]

**Appendix**

Table A1: Full regression results for production function estimates (corresponds with Table 2)

|  | (1) | (2) | (3) | (4) | (5) | (6) |
| --- | --- | --- | --- | --- | --- | --- |
| VARIABLES | POLS | POLS | CRE | CRE | FE | FE |
|  |  |  |  |  |  |  |
| N | 9.329*** | 9.069*** | 8.482*** | 8.214** | 10.31** | 9.797** |
|  | (2.874) | (2.884) | (3.195) | (3.207) | (4.781) | (4.812) |
| N^2 | -0.0141** | -0.0142** | -0.0122** | -0.0123** | -0.0125 | -0.0134 |
|  | (0.00605) | (0.00604) | (0.00613) | (0.00612) | (0.0114) | (0.0110) |
| POXC | 0.484 | 0.474 | 0.491 | 0.480 |  |  |
|  | (0.342) | (0.342) | (0.343) | (0.343) |  |  |
| N*POXC | 0.00440** |  | 0.00432** |  | 0.00545* |  |
|  | (0.00172) |  | (0.00189) |  | (0.00298) |  |
| N*POXC*log(rain) |  | 0.000775** |  | 0.000765** |  | 0.00109** |
|  |  | (0.000325) |  | (0.000356) |  | (0.000525) |
| log(rain) | 2,990*** | 2,998*** | 3,782*** | 3,796*** | 3,853*** | 3,879*** |
|  | (894.1) | (894.1) | (1,060) | (1,060) | (1,139) | (1,137) |
| CV(rain) | -1,357* | -1,356* | -1,207 | -1,204 | -1,890** | -1,867* |
|  | (804.5) | (804.3) | (835.9) | (836.1) | (959.3) | (957.8) |
| P | 1.104 | 1.137 | 1.957 | 1.981 | 10.39 | 10.08 |
|  | (5.521) | (5.517) | (5.801) | (5.796) | (8.888) | (8.826) |
| K | -20.32 | -20.14 | -27.15 | -26.97 | -7.721 | -7.358 |
|  | (32.91) | (32.97) | (33.38) | (33.45) | (30.57) | (31.00) |
| P^2 | -0.0103 | -0.0107 | -0.0136 | -0.0139 | -0.0486* | -0.0476* |
|  | (0.0147) | (0.0146) | (0.0156) | (0.0154) | (0.0264) | (0.0255) |
| K^2 | 0.162 | 0.161 | 0.199 | 0.198 | 0.0871 | 0.0858 |
|  | (0.213) | (0.213) | (0.215) | (0.215) | (0.204) | (0.206) |
| intercropped (=1) | -186.0 | -185.4 | -250.1 | -250.4 | -274.7 | -271.4 |
|  | (169.0) | (169.1) | (272.0) | (271.8) | (291.0) | (290.7) |
| rotation (=1) | -587.1 | -586.6 | -853.0 | -851.2 | -226.3 | -225.3 |
|  | (421.9) | (421.9) | (804.3) | (804.4) | (557.0) | (557.1) |
| manure (=1) | 420.3** | 421.6** | 313.8 | 315.2 | 360.0 | 360.5 |
|  | (182.5) | (182.5) | (232.3) | (232.2) | (238.0) | (237.8) |
| crop residues (=1) | -550.6** | -548.4** | -99.22 | -96.54 | 73.21 | 78.50 |
|  | (249.7) | (249.7) | (363.3) | (363.2) | (389.7) | (389.8) |
| number of weedings | 85.06 | 84.25 | -216.4 | -218.0 | -272.6 | -274.7 |
|  | (157.9) | (157.9) | (213.9) | (213.6) | (218.4) | (218.2) |
| herbicide (=1) | -40.56 | -48.16 | 578.5 | 576.2 | 669.2* | 667.9* |
|  | (427.4) | (425.9) | (402.6) | (401.2) | (351.8) | (351.1) |
| pesticide (=1) | -1,767*** | -1,753*** | -1,723*** | -1,714*** | -1,750*** | -1,722*** |
|  | (496.0) | (496.1) | (405.8) | (407.0) | (424.8) | (424.8) |
| improved seed (=1) | 420.5** | 421.2** | 241.6 | 242.1 | 30.28 | 29.61 |
|  | (195.1) | (195.0) | (243.6) | (243.7) | (261.1) | (261.3) |
| log(plot hectares) | 61.67 | 60.70 | 194.2 | 191.3 | 329.5* | 327.2* |
|  | (97.65) | (97.65) | (168.7) | (168.7) | (167.6) | (167.8) |
| log(farm hectares) | 303.1*** | 302.8*** | 301.7*** | 301.2*** |  |  |
|  | (98.19) | (98.19) | (103.4) | (103.3) |  |  |
| aged 19-23 | -82.43 | -82.92 | -131.2 | -131.5 |  |  |
|  | (582.4) | (582.8) | (602.0) | (602.0) |  |  |
| aged 24-28 | 550.4 | 548.1 | 551.1 | 548.7 |  |  |
|  | (358.8) | (358.8) | (359.9) | (359.8) |  |  |
| aged 29-33 | 376.4 | 377.6 | 344.3 | 345.8 |  |  |
|  | (255.9) | (255.9) | (264.3) | (264.3) |  |  |
| aged 34-38 | 211.8 | 211.9 | 178.7 | 178.9 |  |  |
|  | (234.0) | (234.0) | (237.1) | (237.0) |  |  |
| aged 39-43 | 70.06 | 70.91 | 99.52 | 100.8 |  |  |
|  | (209.3) | (209.8) | (219.3) | (219.7) |  |  |
| female head (=1) | 5.182 | 3.484 | 11.84 | 9.900 |  |  |
|  | (194.7) | (194.5) | (194.0) | (193.8) |  |  |
| household members | 8.393 | 8.333 | 1.633 | 1.528 |  |  |
|  | (26.83) | (26.84) | (26.97) | (26.97) |  |  |
| head education (years) | -1.247 | -1.427 | -2.227 | -2.386 |  |  |
|  | (19.21) | (19.23) | (19.32) | (19.34) |  |  |
| Constant | -15,724*** | -15,769*** | -7,025 | -6,969 | -20,443*** | -20,632*** |
|  | (5,457) | (5,456) | (10,838) | (10,818) | (7,527) | (7,513) |
|  |  |  |  |  |  |  |
| Estimator | POLS | POLS | CRE | CRE | FE | FE |
| Mundlak-Chamberlain controls | no | no | yes | yes | no | no |
| District dummies | yes | yes | yes | yes | no | no |
| Year dummies | yes | yes | yes | yes | yes | yes |
| Observations | 599 | 599 | 599 | 599 | 498 | 498 |
| R-squared | 0.332 | 0.332 | 0.344 | 0.344 | 0.238 | 0.239 |

Notes: The dependent variable in all models is maize yield measured in kg ha^-1^. N = nitrogen; POXC = active carbon; CV = coefficient of variation. Rainfall measured in 10-day periods during the growing season for the survey year. For age dummies, the base category is age 45 and over. Standard errors are cluster robust at the household level. Significance denoted by * (p<0.1), ** (p<0.05) and *** (p<0.01).

Table A2: Production function estimates using total carbon (instead of POXC)

|  | (1) | (2) | (3) | (4) | (5) | (6) |
| --- | --- | --- | --- | --- | --- | --- |
| VARIABLES | POLS | POLS | CRE | CRE | FE | FE |
|  |  |  |  |  |  |  |
| N | 21.85*** | 21.96*** | 20.28*** | 20.26*** | 17.42* | 16.43 |
|  | (4.858) | (4.766) | (4.884) | (4.744) | (9.379) | (10.25) |
| N^2 | -0.0192** | -0.0191** | -0.0172** | -0.0170** | -0.0110 | -0.0102 |
|  | (0.00783) | (0.00767) | (0.00797) | (0.00780) | (0.0131) | (0.0132) |
| carbon | 0.0873*** | 0.0887*** | 0.0874*** | 0.0886*** |  |  |
|  | (0.0208) | (0.0208) | (0.0210) | (0.0210) |  |  |
| N*carbon | -0.00101*** |  | -0.00101*** |  | -0.000692 |  |
|  | (0.000339) |  | (0.000359) |  | (0.000890) |  |
| N*carbon*log(rain) |  | -0.000162*** |  | -0.000161*** |  | -9.19e-05 |
|  |  | (5.25e-05) |  | (5.55e-05) |  | (0.000165) |
| log(rain) | 3,078*** | 3,079*** | 3,787*** | 3,736*** | 3,915*** | 3,875*** |
|  | (884.6) | (885.2) | (1,058) | (1,059) | (1,148) | (1,145) |
| CV(rain) | -1,599** | -1,587* | -1,488* | -1,482* | -2,144** | -2,114** |
|  | (811.0) | (809.0) | (840.8) | (838.8) | (964.5) | (960.8) |
| P | -0.00932 | -0.0747 | 0.802 | 0.801 | 12.41 | 12.41 |
|  | (5.796) | (5.815) | (6.092) | (6.114) | (8.595) | (8.609) |
| K | -25.93 | -26.11 | -30.38 | -30.48 | -9.165 | -9.087 |
|  | (33.00) | (32.75) | (33.21) | (33.01) | (30.13) | (29.82) |
| P^2 | -0.0156 | -0.0150 | -0.0186 | -0.0181 | -0.0592** | -0.0589** |
|  | (0.0170) | (0.0169) | (0.0178) | (0.0178) | (0.0258) | (0.0257) |
| K^2 | 0.196 | 0.194 | 0.219 | 0.217 | 0.0828 | 0.0835 |
|  | (0.210) | (0.209) | (0.211) | (0.210) | (0.200) | (0.198) |
| intercropped (=1) | -186.0 | -185.4 | -250.1 | -250.4 | -274.7 | -271.4 |
|  | (169.0) | (169.1) | (272.0) | (271.8) | (291.0) | (290.7) |
| rotation (=1) | -587.1 | -586.6 | -853.0 | -851.2 | -226.3 | -225.3 |
|  | (421.9) | (421.9) | (804.3) | (804.4) | (557.0) | (557.1) |
| manure (=1) | 420.3** | 421.6** | 313.8 | 315.2 | 360.0 | 360.5 |
|  | (182.5) | (182.5) | (232.3) | (232.2) | (238.0) | (237.8) |
| crop residues (=1) | -550.6** | -548.4** | -99.22 | -96.54 | 73.21 | 78.50 |
|  | (249.7) | (249.7) | (363.3) | (363.2) | (389.7) | (389.8) |
| number of weedings | 85.06 | 84.25 | -216.4 | -218.0 | -272.6 | -274.7 |
|  | (157.9) | (157.9) | (213.9) | (213.6) | (218.4) | (218.2) |
| herbicide (=1) | -40.56 | -48.16 | 578.5 | 576.2 | 669.2* | 667.9* |
|  | (427.4) | (425.9) | (402.6) | (401.2) | (351.8) | (351.1) |
| pesticide (=1) | -1,767*** | -1,753*** | -1,723*** | -1,714*** | -1,750*** | -1,722*** |
|  | (496.0) | (496.1) | (405.8) | (407.0) | (424.8) | (424.8) |
| improved seed (=1) | 420.5** | 421.2** | 241.6 | 242.1 | 30.28 | 29.61 |
|  | (195.1) | (195.0) | (243.6) | (243.7) | (261.1) | (261.3) |
| log(plot hectares) | 55.95 | 57.38 | 157.5 | 161.8 | 305.3* | 310.2* |
|  | (91.45) | (91.64) | (166.1) | (165.1) | (166.2) | (166.5) |
| log(farm hectares) | 297.9*** | 298.5*** | 300.3*** | 302.4*** |  |  |
|  | (94.17) | (94.13) | (98.25) | (98.21) |  |  |
| aged 19-23 | 104.0 | 129.1 | 11.53 | 35.34 |  |  |
|  | (638.9) | (633.7) | (627.4) | (626.5) |  |  |
| aged 24-28 | 583.9 | 580.9 | 592.8 | 589.6 |  |  |
|  | (365.7) | (365.5) | (370.6) | (370.9) |  |  |
| aged 29-33 | 372.1 | 365.4 | 345.3 | 337.5 |  |  |
|  | (258.0) | (258.5) | (268.0) | (268.6) |  |  |
| aged 34-38 | 192.8 | 192.4 | 182.1 | 180.7 |  |  |
|  | (230.0) | (230.5) | (232.3) | (232.9) |  |  |
| aged 39-43 | 101.5 | 94.34 | 137.9 | 129.1 |  |  |
|  | (191.3) | (190.4) | (200.3) | (199.6) |  |  |
| female head (=1) | 25.89 | 24.90 | 38.38 | 38.97 |  |  |
|  | (186.0) | (186.7) | (184.1) | (185.0) |  |  |
| household members | 12.01 | 12.02 | 4.794 | 4.934 |  |  |
|  | (25.35) | (25.32) | (25.52) | (25.50) |  |  |
| head education (years) | -4.513 | -4.334 | -6.742 | -6.724 |  |  |
|  | (18.67) | (18.66) | (18.91) | (18.90) |  |  |
| Constant | -16,890*** | -16,921*** | -11,651 | -12,359 | -20,576*** | -20,355*** |
|  | (5,382) | (5,388) | (10,356) | (10,427) | (7,582) | (7,568) |
|  |  |  |  |  |  |  |
| Estimator | POLS | POLS | CRE | CRE | FE | FE |
| Mundlak-Chamberlain controls | no | no | yes | yes | no | no |
| District dummies | yes | yes | yes | yes | no | no |
| Year dummies | yes | yes | yes | yes | yes | yes |
| Observations | 599 | 599 | 599 | 599 | 498 | 498 |
| R-squared | 0.351 | 0.352 | 0.363 | 0.363 | 0.237 | 0.236 |

Notes: The dependent variable in all models is maize yield measured in kg ha^-1^. N = nitrogen; carbon = total carbon; CV = coefficient of variation. Rainfall measured in 10-day periods during the growing season for the survey year. For age dummies, the base category is age 45 and over. Standard errors are cluster robust at the household level. Significance denoted by * (p<0.1), ** (p<0.05) and *** (p<0.01).

Table A3: Marginal effects from production function estimates using total carbon (instead of POXC)

|  | (1) | (2) | (3) | (4) | (5) | (6) |
| --- | --- | --- | --- | --- | --- | --- |
|  | POLS | POLS | CRE | CRE | FE | FE |
|  |  |  |  |  |  |  |
| N | 12.47*** | 12.68*** | 10.96*** | 11.14*** | 11.11*** | 11.20*** |
|  | (2.670) | (2.690) | (2.875) | (2.880) | (4.280) | (4.318) |
| carbon | 0.0583*** | 0.0586*** | 0.0582*** | 0.0587*** | -0.0197 | -0.0169 |
|  | (0.0191) | (0.0190) | (0.0191) | (0.0189) | (0.0253) | (0.0303) |
|  |  |  |  |  |  |  |
| interaction | N*carbon | N*carbon* log(rain) | N*carbon | N*carbon* log(rain) | N*carbon | N*carbon* log(rain) |

Notes: Table shows partial effects of nitrogen (N) and total carbon from model results in table A2. Units are kg maize per kg N and kg maize per mg kg^-1^ carbon, respectively. Standard errors are cluster robust at the household level. Standard errors are cluster robust at the household level. Significance denoted by * (p<0.1), ** (p<0.05) and *** (p<0.01).
